# In response to “Is solute movement within the extracellular spaces of brain gray matter brought about primarily by diffusion or flow?”

**DOI:** 10.1186/s12987-019-0143-8

**Published:** 2019-07-12

**Authors:** Lori Ray, Jeffrey J. Iliff, Jeffrey J. Heys

**Affiliations:** 1 Chemical and Biological Engineering, Montana State University, Bozeman, MT USA; 2 Department of Anesthesiology and Perioperative Medicine, Oregon Health & Sciences University, Portland, OR USA; 3 Knight Cardiovascular Institute, Oregon Health & Sciences University, Portland, OR USA

## Abstract

In our work, “Analysis of Convective and Diffusive Transport in the Brain Interstitium”, published in this journal (2019, 16:6), we estimate the interstitial superficial velocity by comparison of transport model simulations to published experimental Real-Time Iontophoresis (RTI) data. In the Discussion section, we calculate a value for perfusion rate, or volumetric flow rate per unit mass of tissue, from these fundamental results of superficial velocity. Drs. Hladky and Barrand have proposed an alternative method for choosing the surface area per volume used to calculate perfusion rate from superficial velocity, using our model domain. Their method seems reasonable to us, as does ours. Upon reflection, a range of volumetric flow per unit mass values should have been reported in our paper, 1–40 μL/min-g. The value calculated using Drs. Hladky and Barrand surface area is a likely upper-bound on this range and the value in the paper is a low estimate at the bottom of the range. We are confident in the estimates of interstitial velocity reported in our article, using the assumptions of the model. Peclet (*Pe*) numbers, which compare convective and diffusive transport rates for different molecules, were calculated using the superficial velocity estimates; and we continue to believe these values are correct along with all other major results and conclusions presented in the paper.

We thank Drs. Hladky and Barrand for their careful reading of our paper, insightful comments, and utilization of our work [1].

In our paper, we estimate the interstitial superficial velocity using a porous media transport model of the brain parenchyma [2]. Interstitial superficial velocity is the direct outcome of our model simulation from fundamental transport equations. We are confident in the estimates of interstitial velocity made by comparison of model simulations to published experimental Real-Time Iontophoresis (RTI) data to be accurate, using the assumptions of the model. Peclet (*Pe*) numbers, which compare convective and diffusive transport rates for different molecules, were calculated using the superficial velocity estimates; and we continue to believe these values are correct along with all other major results presented in the paper. The *Pe* calculations show that convection is not an important mechanism of transport for small molecules such as TMA (74 Da), in agreement with Hladky. However, *Pe* calculations show that convective transport may likely be important for molecules larger than 3 kDa (of relevance to neurodegenerative diseases), which have slow diffusivities, made slower by the restrictions of moving through interstitial space. (The apparent diffusivities of TMA and Dextran-3 differ by an order of magnitude.)

We are less confident in our calculation of volumetric flow per gram of tissue. At the request of a reviewer, we attempted to calculate a volumetric flow per unit mass value, or perfusion, from our superficial velocity estimates. Perfusion rate is not a widely used concept in engineering; and a direct conversion from superficial velocity to perfusion rate was not available. However, we understand perfusion is frequently measured experimentally for vascular flow using MRI. Because the perfusion quantity is experimentally measured, we found the conversion from a superficial velocity to perfusion to be difficult as we did not know the details of a typical experiment.

To calculate the volumetric flow rate, one must integrate the superficial velocity over a surface. Small changes to our idealized model, however, have a significant impact on the volumetric flow rate obtained after integration. The perfusion rate we were asked to compare to was calculated from experimental values of whole-brain clearance of sucrose and inulin—a much larger region than our model domain. Therefore, we estimated the perfusion value from our superficial velocity by choosing a flat surface at the midpoint between our idealized banks of arterioles and venules, a square centimeter in a cubic centimeter of tissue, and dividing by a brain tissue density of 1.04 g/cm^3^ approximately 1 cm^2^/g (as quoted by Hladky and Barrand).

The letter from Drs. Hladky and Barrand takes a different approach to the conversion between superficial velocity and volumetric flow rate per gram of tissue [1]. They use a portion of our model domain, between the idealized bank of arterioles and bank of venules, calculating 40 cm^2^/g of tissue. This is a completely reasonable method, but may lead to a higher volumetric flow per unit mass than an experimentally measured value because the cross section of our idealized model domain has all the velocity in a single direction, with no flow in the opposite direction, an unlikely scenario in normal living tissue.

Upon reflection, we should have reported a range of volumetric flow per unit mass values in our paper, 1–40 μL/min-g. The value calculated using Dr. Hladky’s surface area is a likely upper-bound on this range and the value in the paper is a low estimate at the bottom of the range. In addition, we should have questioned the appropriateness of comparing a local velocity to a volumetric flow rate for clearance from the entire brain. The two values have different purposes. A system-wide volumetric clearance rate reflects the net effect of transport processes averaged across the brain and may include many processes in addition to local diffusion and convection. The goal of our local investigation was to understand the effect of a potential convective velocity in the brain interstitium on local molecular transport, relative to diffusion-only. Our work was validated with RTI experimental data, which characterize brain transport on the scale of < 1 mm^3^.

Thank you again Drs. Hladky and Barrand for your interest in our work and initiating this discussion.

## Data Availability

Not applicable.

## Abbreviations

*Pe*: Peclet number
RTI: real-time iontophoresis
TMA: tetramethylammonium
